# Pneumothorax by Penetrating Endplate Screw for Diffuse Idiopathic Skeletal Hyperostosis-Related Thoracolumbar Fracture

**DOI:** 10.7759/cureus.33440

**Published:** 2023-01-06

**Authors:** Kentaro Yamada, Yusuke Hori, Toshitaka Yoshii, Shunki Iemura, Atsushi Okawa

**Affiliations:** 1 Department of Orthopedics, Tokyo Medical and Dental University, Tokyo, JPN; 2 Department of Orthopedic Surgery, Osaka Metropolitan University, Osaka, JPN; 3 Department of Orthopedics, PL Hospital, Tondabayashi, JPN

**Keywords:** trauma, spine, surgery, vertebral fracture, thoracolumbar, surgical complication, pedicle screw, penetrating endplate screw, diffuse idiopathic skeletal hyperostosis (dish), pneumothorax ptx

## Abstract

Pneumothorax is a rare surgical complication in spinal surgery with thoracic pedicle screws. The penetrating endplate screw (PES) technique has been developed as a strong alternative spinal anchor to conventional pedicle screws for diffuse idiopathic skeletal hyperostosis (DISH). We present an intraoperative pneumothorax without deviation to the thoracic during the maneuver of the PES.

A 56-year-old male who presented with non-union of DISH-related T12 vertebral fracture underwent T12 kyphoplasty and T10-L2 posterior fixation using the PES technique. The left pneumothorax was developed postoperatively without screw deviation to the thorax throughout screw insertion. Postoperative CT suggested that a displaced rib head by the lateral misposition of the screw at the inserting point and the pedicle level might injure the pleura. Spine surgeons should know that the lateral insertion of PES has a potential risk for pneumothorax by the displacement of the rib head because of screw trajectory from caudal to cranial apart from conventional pedicle screw.

## Introduction

The pedicle screw is a wide-spread spinal anchor that enables control of the instrumented segments and rigid internal immobilization in cases of spinal fixation surgery [1]. The pedicle screw at the thoracic segments is also a standardized method for stabilization or correction for various spinal disorders nowadays, although pneumothorax was reported as a rare complication of the screw placement [2].

Diffuse idiopathic skeletal hyperostosis (DISH) is a non-inflammatory skeletal disease characterized by calcification and ossification of spinal ligaments and entheses [3]. Vertebral fractures related to DISH sometimes become problematic for delayed diagnosis [4], demonstrate spinal instability [5], and need surgical intervention [6]. DISH-related vertebral fractures need stronger fixation even using pedicle screws than non-DISH fractures because of significant fracture site instability with a long lever arm due to the immobilized spinal column by DISH [7]. The penetrating endplate screw (PES) technique has been recently developed for cases with DISH as an alternative pedicle screw, which provides more strong biomechanical strength compared with the conventional pedicle screw and allows less-invasively reduction of instrumented levels [8,9]. In this report, we present a case that showed pneumothorax caused by the lateral insertion of the thoracic PES, review the relevant literature, and discuss the reason for this complication.

## Case presentation

**Figure 1:**
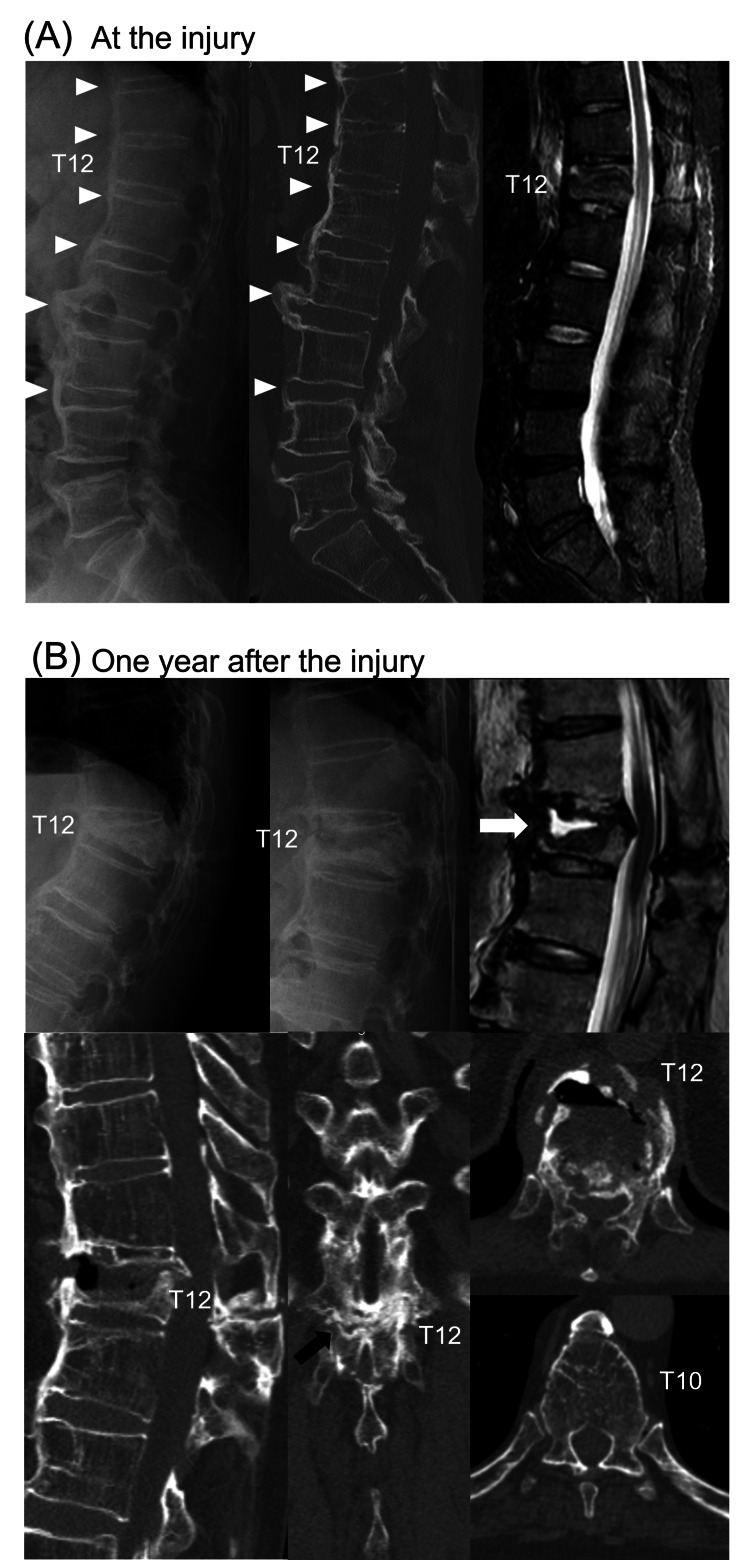
Preoperative radiological findings. The images show (A) radiological findings at the injury. T12 vertebral fracture was not diagnosed by x-ray (left) or CT (center), but by MRI (right). Ossification by DISH was observed between the thoracic segments and L4 (triangle). (B) Radiological findings after one year from the injury. X-rays showed inter-vertebral instability of T12 between the standing position (upper left) and the supine position (upper center). MRI showed a fluid collection at T12 (upper left, white arrow). CT images showed an anterior huge intervertebral cleft and transverse lamina fracture at T12 (black arrow). DISH: diffuse idiopathic skeletal hyperostosis; CT: computed tomography; MRI: magnetic resonance imaging

A 56-year-old male under a prednisolone prescription of 15 mg per day for pemphigoid fell to the floor. T12 vertebral fracture was not diagnosed by the initial radiological examinations, x-rays and computed tomography (CT), but was diagnosed by magnetic resonance imaging (MRI) (Figure 1A). DISH was observed between thoracic segments and L4. Conservative treatment by elastic orthosis was administrated. However, his low back pain had prolonged, and the T12 vertebral fracture developed non-union one year after the injury (Figure 1B). CT images illustrated an anterior huge intervertebral cleft and transverse lamina fracture at T12, which indicated the AO spine thoracolumbar spine injury classification B3 implicating anterior tension band injury.

**Figure 2:**
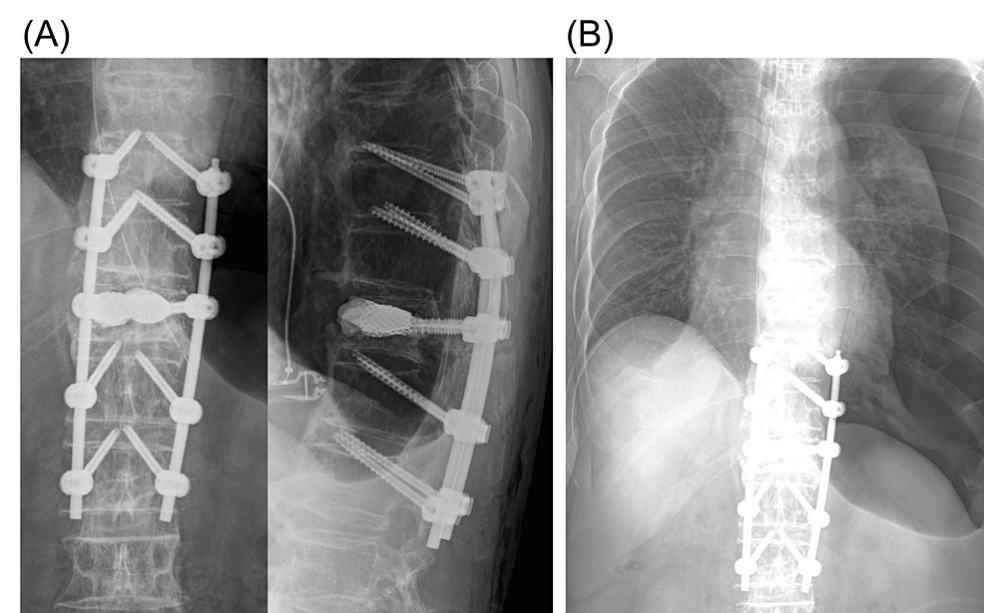
Postoperative x-rays of the patient. The images show (A) kyphoplasty at T12 and T10-L2 posterior fixation using PES was performed by the percutaneous technique. (B) Pneumothorax at the left side could be observed in the postoperative radiograph by adjusting the window level to the lung condition. PES: penetrating endplate screw

T12 kyphoplasty and T10-L2 posterior fixation using the PES technique (VBS and EXPEDIUM VERSE; DePuy Synthes Spine, Inc., Raynham, MA) was performed percutaneously under single fluoroscopy without the navigation system (Figure 2A). All screws were inserted by one-shot placement in all maneuvers of the percutaneous technique. Decreased blood pressure was observed following thoracic screw insertion, however, the anesthesiologist did not alert the surgeons because blood pressure was recovered by administration of intravenous phenylephrine. After confirmation of postoperative x-ray by the attending spine surgeons who had 10- and five-year experience in spine surgery (Figure 2A), the anesthesiologist extubated and the patient returned to the normal ward. None of the surgeons or the anesthesiologist recognized pneumothorax in the operating room, although pneumothorax could be detected retrospectively from the postoperative x-ray by adjusting the window level to the lung condition (Figure 2B).

**Figure 3:**
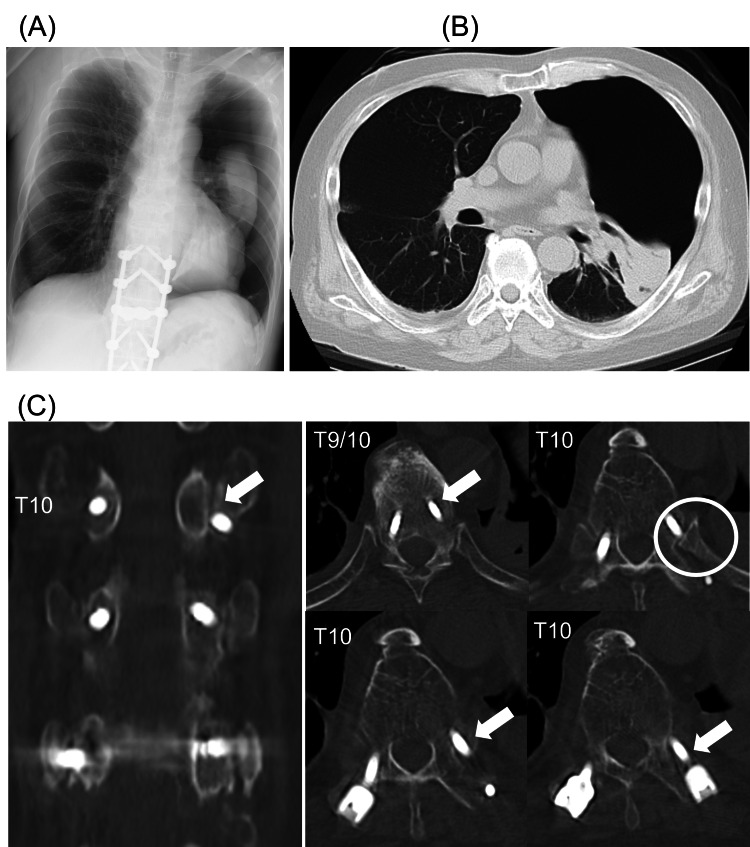
Radiological findings two days after surgery. Pneumothorax on the left side was observed in the chest x-ray (A) and the chest CT (B). Postoperative spine CT (C) showed that the left screw at T10 was placed lateral to the pedicle but caught vertebral body (arrow). The rib head at left T10 displaced to the anterior compared with the right side (circle). CT: computed tomography

The patient complained of slight dyspnea two days after surgery. SpO_2_ indicated 90% even under a simple oxygen mask with a flow rate of 4 L/min. Pneumothorax of the left lung was noticed by chest x-ray (Figure 3A) and CT (Figure 3B). CT also showed that the left side T10 screw was set laterally to the pedicle but caught the vertebral body (Figure 3C). There was no deviation of the screw to the thorax. The rib head of the left T10 was laterally and anteriorly displaced (Figure 3C, circle).

Chest drainage was performed for the pneumothorax immediately by the general surgeon. The left lung was re-expanded after chest drainage. The drainage tube was removed after five days. The patient was discharged after three weeks after the surgery without any other complications and recurrent pneumothorax. Bone union of T12 was confirmed at six months after surgery without any instrument failure.

## Discussion

Pneumothorax is occurred by injuring both the parietal and visceral pleura at the surgery. Under mechanical ventilation, it easily develops tension pneumothorax which is a life-threatening complication [10]. Among patients receiving assisted ventilation, increased intrapleural pressure throughout the respiratory cycle produces an immediate and marked decrease in cardiac venous return, which likely frequently leads to hypotension and may result in cardiac arrest. Therefore, unnoticed pneumothorax during surgery under general anesthesia with mechanical ventilation is a devastating complication.

Pneumothorax has been reported as one of the surgical complications of the conventional thoracic pedicle screw [2], however, the incidence of pneumothorax by the conventional thoracic pedicle screw is very low, only in a few case reports [11,12]. Suk et al. reported one postoperative pneumothorax that required chest drainage after the thoracotomy in their review of complications among 462 patients and 4604 thoracic pedicle screws in surgeries for spinal deformity [11]. Viswanathan et al. reported a case of intraoperative pneumothorax by “in-out-in” thoracic pedicle screw placement [12]. The “in-out-in” technique, extra-pedicular screw placement or pedicle/rib screw fixation, is a useful salvage technique with pull-out strength of more than 70% of that of intrapedicular screws when intra- or transpedicular screw placement is not possible because of small pedicle width or safety reason. They speculated that the pneumothorax in their case occurred by a ball-tip probe via a defect in the costotransverse ligament and capsule of the costovertebral joint when the “in-out-in” screw placement.

**Figure 4:**
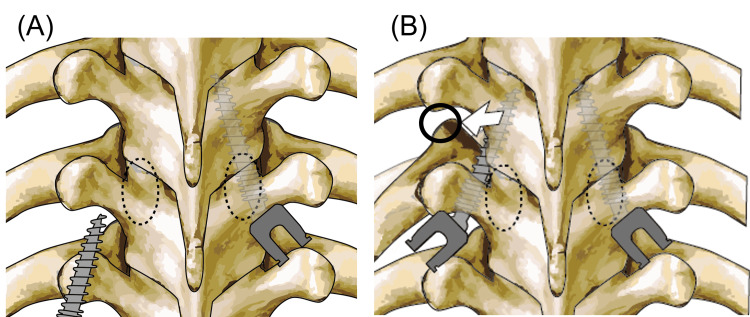
Schema of the reason for pneumothorax by PES insertion. The images show (A) the left side screw was inserted from the lateral of the pedicle wall compared ideal PES placement of the right side. (B) The left rib head was pushed out to the lateral and anterior direction by the screw placed at the cranial of the pedicel level. The anteriorly displaced rib head potentially injured the partial pleura and lung (circle). PES: penetrating endplate screw

The actual cause of pneumothorax in this case remains unclear. The maneuver of percutaneous screw insertion was performed in one-shot placement for all screws percutaneously and there was no screw deviation to the thorax in the postoperative CT. One possible reason was injury of the pleura and lung by the displaced rib head due to lateral extrapedicular placement of the PES. Postoperative CT showed rib head of the left T10 was laterally and anteriorly displaced (Figure 3C, circle), compared with preoperative CT (Figure 1B). The lateral and cranial extrapedicular placed screw might disrupt the costotransverse ligament/the costovertebral joint capsule and push the rib head to the anterior and lateral (Figures 4A, 4B). The pleura and lung were potentially injured by the displaced rib head combined with the effect of tissue fragility due to long-time administration of prednisolone [13].

Although the percutaneous screw placement technique using fluoroscopy was reported as an acceptable technique with a lower deviation/complication rate than the conventional open free-hand technique, the direction of the screw misplacement in the percutaneous technique was reportedly more lateral than medial pedicle violation [14]. It arises that screw placement depends on the AP view of the fluoroscopy without direct confirmation of the anatomy. In this case, the entry point of the PES at the left T10 was misplaced laterally. If this screw had been a conventional pedicle screw, it would have been set in the “in-out-in” trajectory even if the entry point deviated laterally and would not have caused a pneumothorax. Apart from "in-out-in" technique for the conventional pedicle screw, "in-out-in" of PES has a potential risk for the displacement of the rib head because of the screw trajectory from the caudal to the cranial of the pedicle, just adjacent rib head at the pedicle level (Figures 4A, 4B). Therefore, "in-out-in" of PES has a potential risk for pneumothorax, pneumothorax might be a rather specific complication of the PES technique than the traditional pedicle screw. Although this case, fortunately, did not develop tension pneumothorax, spine surgeons should take attention to lateral deviation in cases using the PES technique. The use of dual-planar fluoroscopy, intraoperative CT, or the navigation system would reduce the risk of pneumothorax by the PES technique.

## Conclusions

Pneumothorax is a rare complication of the conventional thoracic pedicle screw. However, the PES which trajectory from the caudal to the cranial of the pedicle has a greater risk of pneumothorax than the conventional screw in the case of lateral deviation even without direct injury of the pleura by the instrument. Because pneumothorax under ventilation is a life-threatening complication, spine surgeons should prevent lateral deviation in cases using the PES technique.
